# DABCO bond cleavage for the synthesis of piperazine derivatives†

**DOI:** 10.1039/c9ra07870c

**Published:** 2019-11-08

**Authors:** Azim Ziyaei Halimehjani, Elham Badali

**Affiliations:** Faculty of Chemistry, Kharazmi University 49 Mofateh St. 15719-14911 Tehran Iran ziyaei@khu.ac.ir

## Abstract

The applications of DABCO (1,4-diazabicyclo[2.2.2]octane) in the synthesis of piperazine derivatives including biologically active compounds *via* C–N bond cleavage are investigated in this review. Different reagents such as alkyl halides, aryl(heteroary) halides, carboxylic acids, diaryliodonium salts, tosyl halides, activated alkynes, benzynes *etc.* were applied for the preparation of the corresponding quaternary ammonium salts of DABCO, which are very good electrophiles for various nucleophiles such as phenols, thiophenols, thiols, alcohols, aliphatic and aromatic amines, sulfinates, phthalimide, indoles, NaN_3_, triazole and terazoles, NaCN, enols and enolates, halides, carboxylic acid salts *etc.* Besides preactivated DABCO salts, the *in situ* activation of DABCO in multicomponent reactions is also an efficient tactic in synthetic organic chemistry for the diversity oriented synthesis of drug-like piperazine derivatives.

## Abstract 2

The applications of DABCO (1,4-diazabicyclo[2.2.2]octane) in the synthesis of piperazine derivatives including biologically active compounds *via* C–N bond cleavage are investigated in this review.

## Graphical abstract

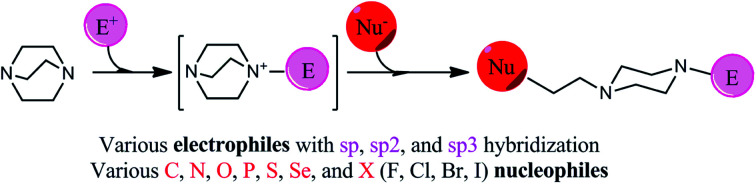

## 1. Introduction

**Fig. 1:**
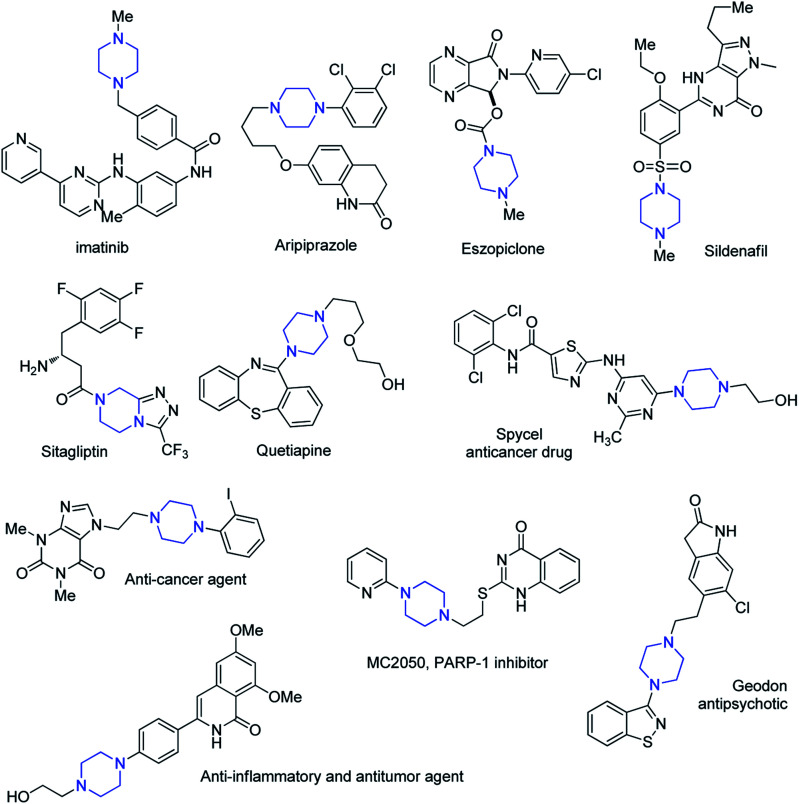
Selected marketed drugs or drug candidates containing piperazine motif.

Heterocyclic rings show rich chemistry with a wide range of applications in a variety of fields such as organic and medicinal chemistry, pharmaceutical chemistry and industry.^1^ A great variety of heterocyclic compounds are presented in the structure of several drugs and renewable resources.^2^ Approximately half of the top 25 best-selling pharmaceuticals from the year 2014 contain nitrogen heterocyclic scaffolds with more than 50 billion USD in annual revenue.^3^ In addition, FDA databases reveal that nearly 60% of unique small-molecule drugs contain a nitrogen heterocycle.^4^ Among various nitrogen containing heterocyles, piperazine is very important building block in the structure of many compounds with widespread applications in different fields such as material, agrochemical, and medicinal chemistry. Piperazine is essential core in drug design and statistical substructure analysis revealed that it is the third most commonly used N-heterocycle (ranked after piperidine and pyridine) in small-molecule pharmaceuticals.^5^ Currently MDDR (MDL@Drug Data Report) database contains over 11 800 structures bearing the piperazine moiety. These compounds have shown various biological activities such as anti-fungal,^6^ anti-depressant,^7^ anti-malarial,^8^ anti-migraine,^9^ anti-diabetic,^10^ anti-aggregating,^11^ anti-tumor,^12^ anti-inflammatory,^13^ anti-obesity,^14^ and cardiovascular^15^ activities. The structure of some of the marketed drugs or drug candidates containing piperazine motif is shown in Fig. 1. Some of them such as Imatinib, Aripiprazole, Eszopiclone, Sitagliptin, Quetiapine and Sildenafil are within the top 100 best-selling drugs.^4^ In most of these compounds, piperazine scaffold is mainly used as a linker between two portions of a drug or as mediator for tuning the physicochemical properties of drugs. Similarly, most of the piperazines isolated from natural products are unsubstituted at any of their carbon atoms. Besides biological activities, piperazine derivatives have found widespread applications in liquid crystals,^16^ metal–organic frameworks,^17^ coordination chemistry,^18^ coating, adhesives and sealing materials,^19^ self-assembled monolayer in electronic devices,^20^ novel charge-transfer polymers in solar cells,^21^ antistatic agents and vulcanization accelerators.^22^

**Fig. 2:**
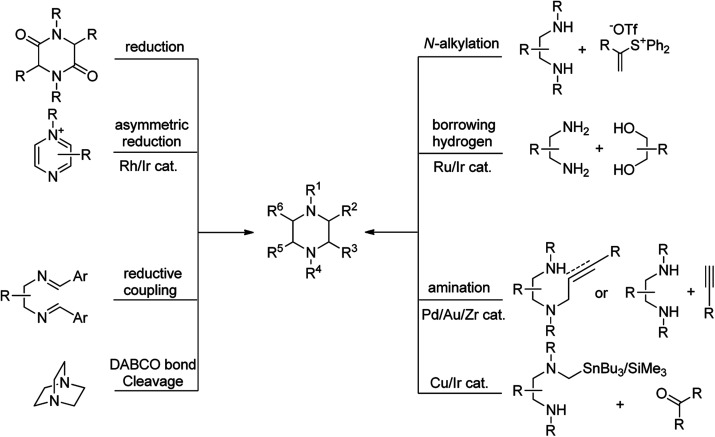
General synthetic strategies toward piperazine scaffold.

The biological and industrial importance of piperazine derivatives encouraged chemists to find general and efficient approaches for the synthesis of these compounds. Various methods are available for the synthesis of piperazine derivatives in literature, most of them rely on cyclisation procedure. Novel synthetic approaches toward piperazine ring can be divided to the following main categories: (1) reduction of (di)ketopiperazine (di)ketopiperazine are generally synthesized from the amino acids or 1,2-diamines and other readily available starting materials, (2) *N*-alkylation of diamines with electrophiles including α-halocarbonyl compounds, vinyl sulfonium salts, vinyl selenones, and *etc.*, (3) catalytic or chemical reduction of pyrazines, pyrazinium salts, and pyrazine-*N*-oxides, (4) cyclocondensation of amines with alcohols *via* borrowing hydrogen strategy, (5) intramolecular reductive coupling of 1,2-diimines, (6) intermolecular or intramolecular amination of alkenes or alkynes, (7) DABCO bond cleavage, and (8) transition-metal-catalyzed condensation of stannyl (silicon) amine reagents with carbonyl compounds (Fig. 2). Other methods such as dimerization of aziridines [4 + 2]-cycloaddition of 1,2-diamines with allenes and alkynes, post-Ugi reaction, rearrangement of spiro compounds, and C–H functionalization of piperazine ring are also well investigated in recent years.^23^ Among the reported methods, DABCO bond cleavage is one of the most efficient and simple approaches for the synthesis of functionalized piperazines without affecting the carbons of piperazine ring. While most of the marketed or drug candidates contain simple piperazine ring in their structure, this strategy can be further utilized for the development of novel biologically active compounds in future.

**Fig. 3:**
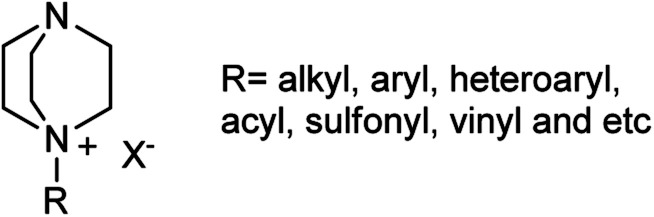
DABCO-quaternary salts.

According to the best of our knowledge, a review covering the synthesis of piperazine derivatives *via* DABCO bond cleavage is not available in the literature. For this purpose, this review serves as a comprehensive overview of published papers in the time range between 1962 until today for the synthesis of functionalized piperazine derivatives *via* DABCO bond cleavage. While preparation of DABCO based quaternary salts (Fig. 3) is the key step for the activation of C–N bond in DABCO for cleavage, in this review paper, the papers are categorized according to the type of activating agents such as alkyl halides, aryl(heteroaryl) halides, carboxylic acids, diaryliodonium salts, tosyl halides, activated alkynes, benzynes and *etc.*

## 2. Alkyl halides as activating agents

**Scheme 1:**
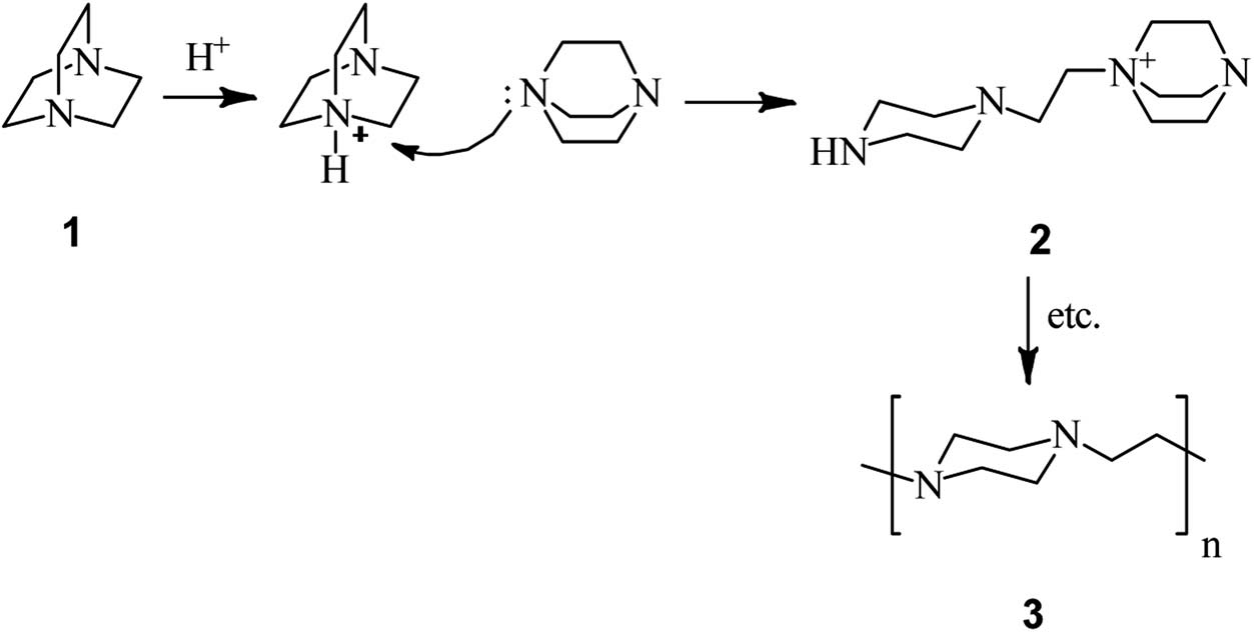
Synthesis of poly-1,4-ethylenepiperazine from DABCO.

The first report on DABCO bond cleavage is came back to 1962 by H. K. Hall.^24^ He showed that by heating 1,4-diazabicyclo[2.2.2]octane 1 at 200 °C for 10.7 h in the presence of a catalytic amount of benzenesulfonic acid, the corresponding poly-1,4-ethylenepiperazine 3 can be obtained in 96% yield. He concluded that the reaction proceeded *via* nucleophilic attack of the nitrogen of one DABCO on the protonated 2 or alkylated 3 form of another (Scheme 1).

**Scheme 2:**
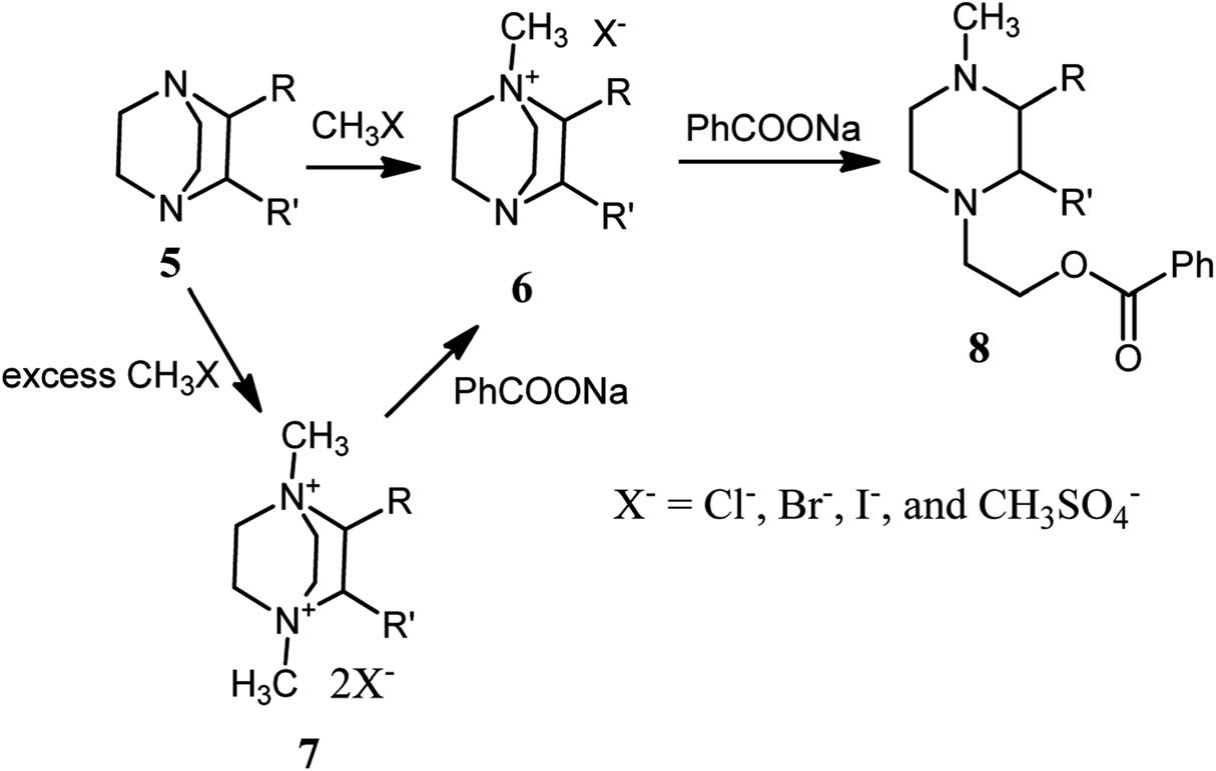
Synthesis of mono- or bisquaternary salts of DABCO and their ring opening with sodium benzoate.

In 1982, Vysochin and Shishkin^25^ reported the reaction of 2- or/and 3-substituted DABCO derivatives 5 with methylating agents such as methyl iodide, methyl benzoates and dimethyl sulfate for the preparation of the corresponding mono- or bisquaternary salts 6 and 7. The monoquaternary salts 6 can be converted to the corresponding piperazine derivatives by reacting with a nucleophile such as sodium benzoate at 130–140 °C. The bisquaternary salts 7 were converted to the corresponding monoquaternary salts 6 prior to DABCO bond cleavage with nucleophile to provide the piperazine derivatives 8 (Scheme 2).

**Scheme 3:**
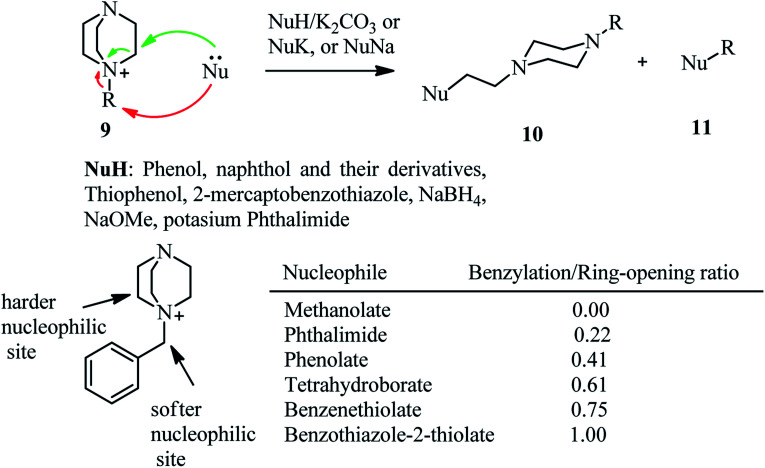
C–N bond cleavage of DABCO quaternary salts with various nucleophiles.

**Scheme 4:**
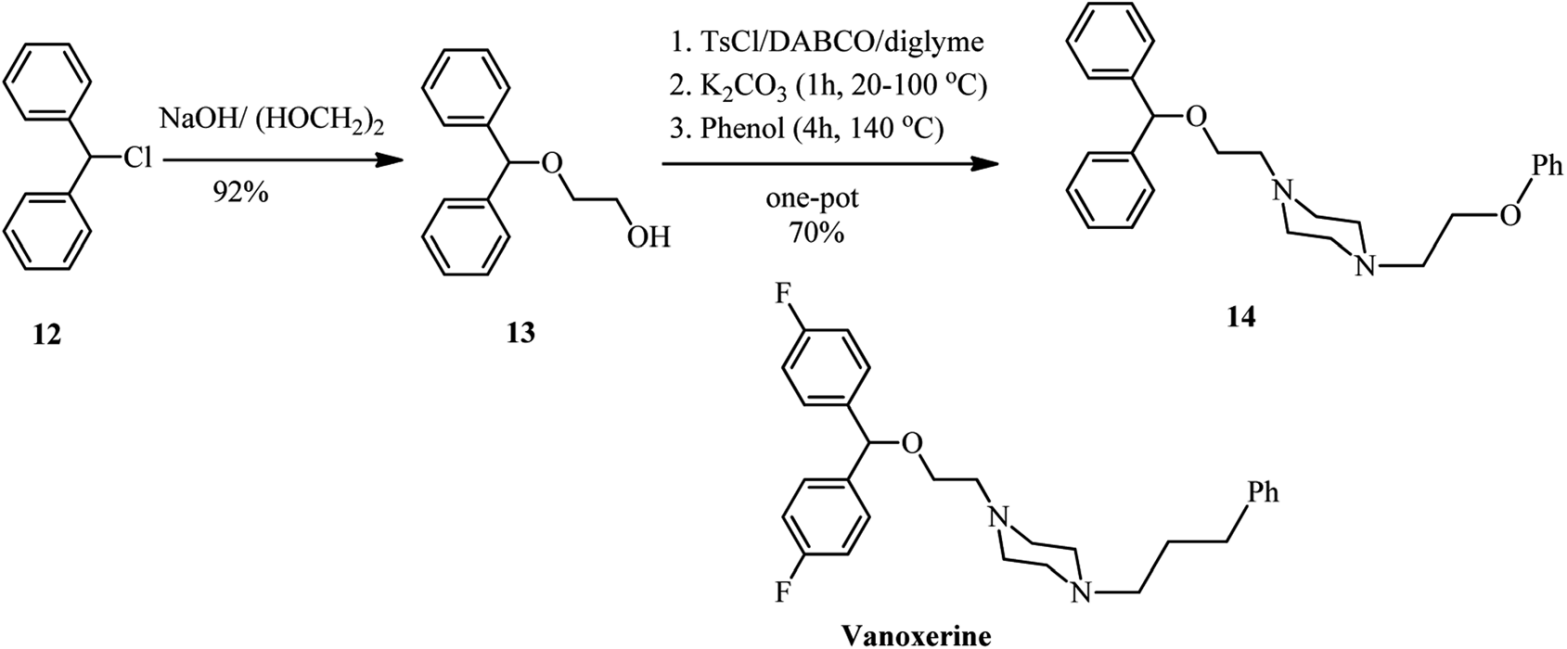
Synthesis of ether isosteres of Vanoxerine *via* DABCO bon cleavage.

Recently, Kocevar *et al.*^26^ have investigated the reaction of quaternary salts of DABCO 9 with phenols and related nucleophiles to prepare 1-alkyl-4-(2-phenoxyethyl) piperazines and related derivatives 10. The reactions were performed in polyethyleneglycol (PEG) or diglyme at high temperatures. Various nucleophiles such as phenols, thiophenols, potassium phthalimide, sodium methoxide, and benzothiazole-2-thioate were applied in DABCO ring opening reactions to give moderate to high yields of piperazine derivatives 10. The regioselectivity and mechanism of the reaction is depending on the nature of alkyl halides on the DABCO. Generally, the alkylation reaction competes with the DABCO ring opening reaction. The ratio of products is depending on the hard–soft properties of the nucleophiles and electrophiles (HSAB theory) and the steric hindrance of the alkyl halides. For example, while in the reaction of DABCO salts containing simple alkyl groups with oxygen nucleophiles such as sodium methoxide and potassium phenoxide, the DABCO ring-opening products 10 were obtained with high to excellent regioselectivity, by using benzyl group as alkylating agents in DABCO salt and soft nucleophiles such as benzenethiolate and benzothiazole-2-thiolate, the benzylation adducts 11 were obtained as major product. The results of the reaction of 9 (R = benzyl) with various nucleophiles is tabulated in Scheme 3. In addition, this strategy was successfully applied for the synthesis of compounds 14 from 12 in four steps, a prototype of a series of potential dopamine reuptake inhibitors, which can be considered as ether isosteres of Vanoxerine (Scheme 4). In this report, alkyl tosylate was applied instead of alkyl halides for DABCO bond cleavage.

**Scheme 5:**
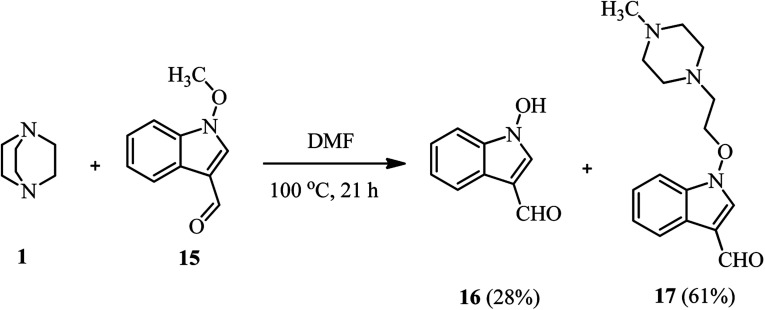
DABCO bond cleavage *via* the reaction of DABCO with 1-methoxy-indole-3-carbaldehyde.

In addition to alkyl halides and tosylates, Somei *et al.* reported that while the reaction of DABCO 1 with 1-methoxy-indole-3-carbaldehyde 15 in DMF/H_2_O (3 : 1, v/v) at 100 °C afforded the corresponding demethylation product 16 in quantitative yield, by performing the same reaction in DMF without a proton source, the piperazine compound 17 can be obtained as major product in 61% yield (Scheme 5).^27^

## 3. Aryl(heteroary) halides as activating agents

**Scheme 6:**
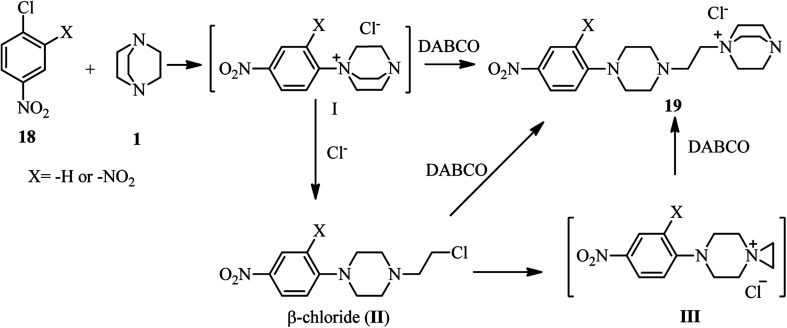
Reaction of nitrochlorobenzenes with DABCO.

The first report on using aryl halides for the preparation of DABCO salts is published by Ross *et al.* in 1963.^28^ They investigated the reactions of nitrochlorobenzenes 18 with DABCO 1 (Scheme 6). They have shown that the only product is the salt 19 and the corresponding salt I was not observed. They confirmed that the reactions are second order; first order in the chloride and first order in the amine and the formation of the intermediate I is the rate-determining step. They concluded that the reaction further proceeded *via* intermediate I, followed by the direct attack of another DABCO to form 19 or attack of the chloride to the methylene group of DABCO salt I to form II, and subsequently S_N^2^_ displacement of the chloride in II by another equivalent of DABCO molecule to form final product 19. In addition, they have shown that by using 2,4-dinitrochlorobenzene in the reaction with DABCO, depend on the ratio of the starting materials, the corresponding salt 19 (in the presence of excess of DABCO) or β-chloride compound II (in the presence of excess 2,4-dinitrochlorobenzene) can be obtained. They proposed that β-chloride II is converted to 19 *via* direct displacement or *via* intermediate III.

**Scheme 7:**
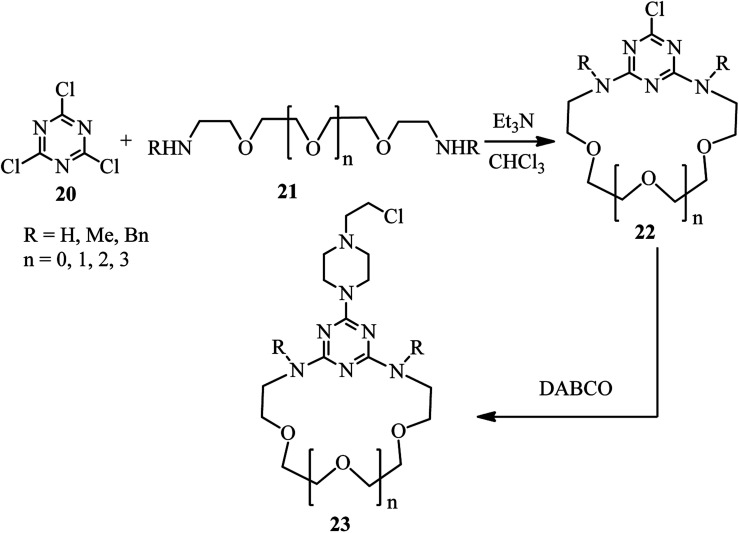
Synthesis of piperazine derivatives containing aza crown ethers of various ring sizes.

Research in this area was stopped for more than 30 years until 1996. In this year, Lutze and coworkers described that the piperazine derivatives containing aza crown ethers of various ring sizes 23 can be prepared by DABCO ring opening strategy in two steps (Scheme 7).^29^ Reaction of trichlorotriazines 20 with PEG-based diamines 21 in the presence of Et_3_N as catalyst and chloroform as solvent afforded the corresponding aza crown ethers 22 in high yields. Reaction of these aza crown ethers 22 with DABCO 1 as the third component activated the methylene positions in DABCO for ring opening reaction, followed by nucleophilic attack by Cl^−^ to afford the corresponding piperazine products 23.

**Scheme 8:**
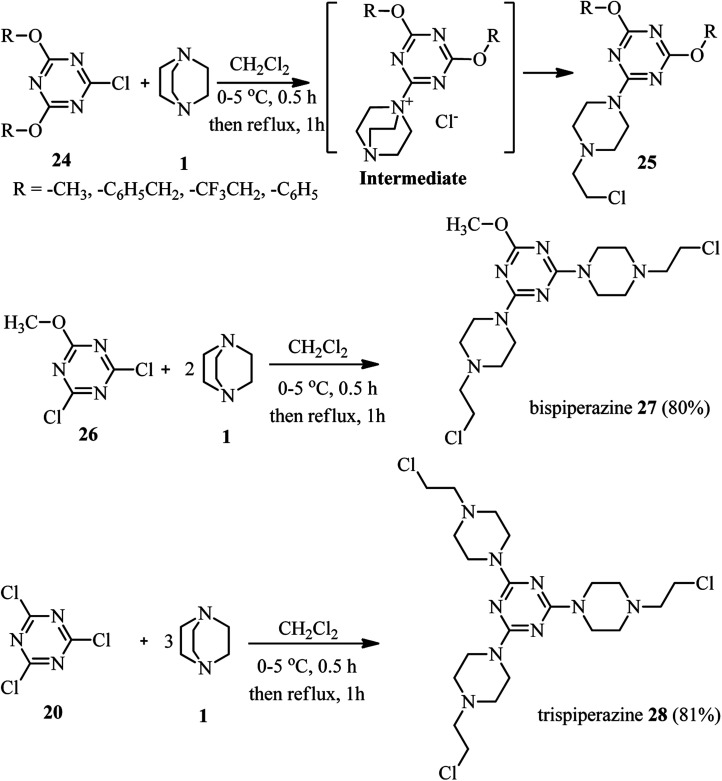
Synthesis of piperazines *via* the reaction of chlorotriazines with various equivalents of DABCO.

In a similar study, Kolesinska research group reported that the reaction of 2,4-bis-dialkoxy(aryloxy)-1-chlorotriazines 24 with 1 leads to the formation of 2-chloroethylamino fragment attached to 1,3,5-triazine *via* a piperazine ring (compound 25).^30^ In addition, they concluded that reaction of 2-methoxy-4,6-dichloro-1,3,5-triazine 26 or trichlorotriazine 20 with excess amount of DABCO afforded the corresponding triazine backbones 27 or 28 with two or three piperazines (Scheme 8). Among the synthesized compounds, the strongest inhibition of proliferation for tumour cells was observed for triazines with single chloroethylamino fragment.

**Scheme 9:**
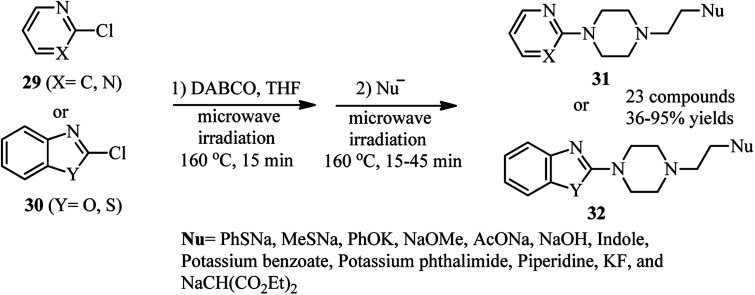
Microwave-assisted DABCO bond cleavage using heteroaryl chlorides.

In 2007, Wang and coworkers reported a highly efficient, two-step procedure for the synthesis of 4-substituted 1-heteroarylpiperazines 31 or 32*via* microwave heating of heteroaryl chlorides 29 or 30 with DABCO and a nucleophile at 160 °C (Scheme 9).^31^ Various heteroaromatics including chloropyrimidines, 2-chloropyridines with electron withdrawing groups, 2-chlorobenzoxazole, 2-chlorobenzothiazole, and 4-chloro-2-phenylquinazoline were applied successfully in this protocol. In addition, a range of nucleophiles including the salt of phenol, methylmercaptan, thiophenol, alcohols, carboxylic acids, phthalimide, indole, diethylmalonate, KF and NaOH were used in this protocol to afford the corresponding piperazines in moderate to excellent yields.

**Scheme 10:**
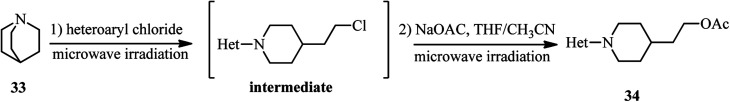
Microwave assisted quinuclidine C–N bond cleavage.

In addition, the same group developed this technology for the synthesis of *N*-heteroaryl-4-(2-chloroethyl)piperidines 34 by using quinuclidine 33 instead of DABCO and their subsequent nucleophilic displacement by sodium acetate (Scheme 10).^32^

**Scheme 11:**
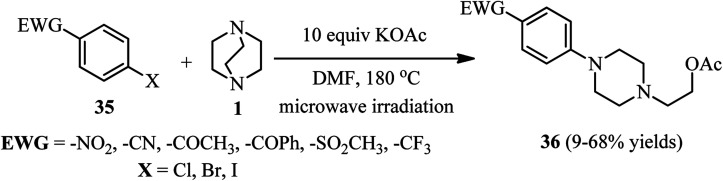
Microwave-assisted DABCO ring opening using *p*-substituted halobenzenes.

Microwave-assisted one-pot three-component synthesis of 1-(*para*-substituted-aryl)-4-(2-acetoxyethyl)piperazines 36 is further developed by Gladstone *et al.*^33^ They have shown that mixing of a para-substituted halobenzene 35 (1 equiv.), DABCO 1 (2 equiv.), and potassium acetate (10 equiv.) in DMF in a microwave reactor and irradiating the mixture to reach 180 °C for 1–4 h, the piperazine adducts 36 can be obtained in 9–68% isolated yields (Scheme 11). Various electron-withdrawing groups such as –NO_2_, –COCH_3_, –CN, –COPh, –SO_2_CH_3_, and –CF_3_ can be applied on the *para*-position of the aryl halide. Carboxylic acid and ester are not suitable groups for the activation of aryl halide toward S_N_Ar reaction. In addition, using 3-chloro-2-cyanothiophene instead of aryl halide gave moderate yields in this protocol. By using 4-bromo-3-chloronitrobenzene, the S_N_Ar reaction of DABCO was occurred regioselectively on the bromide position. By using other nucleophiles such as potassium phthalimide, potassium cyanide, potassium hydroxide, and sodium methoxide instead of potassium acetate, only the potassium phthalimide gave similar yields and no product was detected with other nucleophiles.

**Scheme 12:**
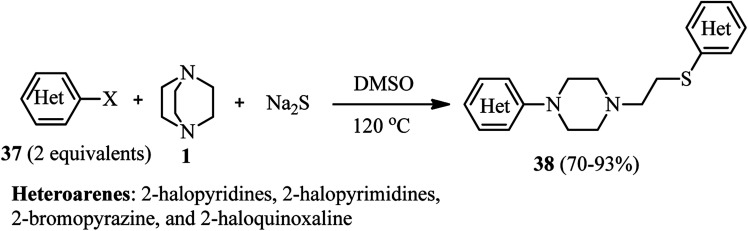
Reaction of azaarene halides, DABCO, and Na_2_S for synthesis of piperazine derivatives.

Efficient protocols for the synthesis of potentially drug-like compounds containing amine, azaarene, thioether, or phenol ether functionalities were introduced by Zhu *et al.*^34^ They have shown that the reaction of various azaarene halides 37 (2 equiv.), DABCO 1, and Na_2_S (1 equiv.) in DMSO at 120 °C afforded the corresponding 4-substituted 1-heteroarylpiperazines 38 in high to excellent yields. While azaarene fluorides were applied as well as bromides and chlorides in this protocol, they indicated that the leaving group does not have significant effect on the reactivity in this protocol (Scheme 12).

**Scheme 13:**
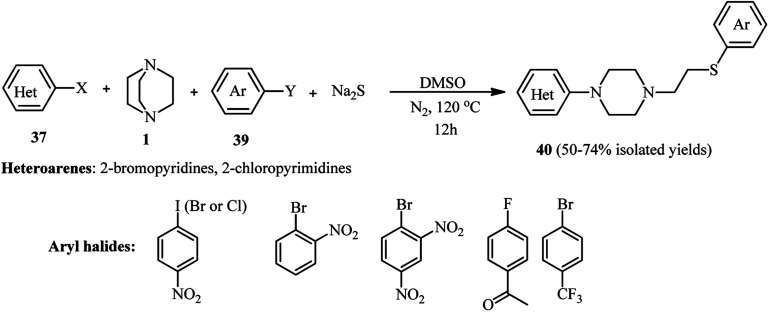
One-pot four-component reaction for the synthesis of piperazine derivatives.

Encouraged by these results, they also developed a one-pot four-component protocol for the synthesis of substituted piperazines 40 using an azaarene halide 37, DABCO 1, an activated aryl halide 39, and Na_2_S (Scheme 13). While 2-bromopyridines were tolerated well in this multicomponent protocol, the one-pot four-component reaction of aryl halides with 2-chloropyrimidines, DABCO, and Na_2_S did not proceed, and only the three-component products 38 were obtained. According to the author's statement, it is due to the higher reactivity of 2-chloropyrimidines toward Na_2_S than activated aryl halides. To overcome this drawback, a two-step sequential procedure, in which Na_2_S and activated aryl halides are pre-mixed prior to the addition of chloropyrimidine and DABCO, was applied and afforded the desired 4-component product in high yields. Fortunately, by developing this two-step sequential reaction process, the structure of the products can be simply tuned by changing the reactant-addition sequence.

**Scheme 14:**
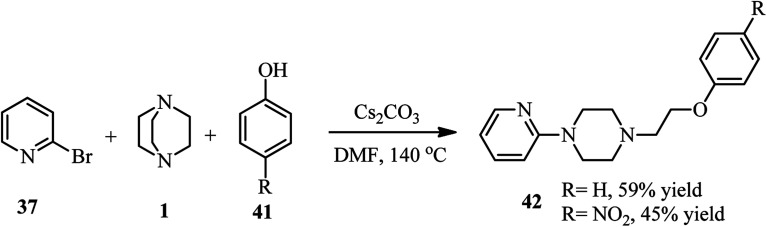
Cs_2_CO_3_-catalyzed DABCO bond cleavage using 2-bromopyridine and phenols.

Furthermore, they have developed that besides the aryl thiolate, phenolates 41 can also be utilized as nucleophiles for the three component reaction. They have shown that reaction of phenols 41 with DABCO 1 and 2-bromopyridine 37 in the presence of Cs_2_CO_3_ at 140 °C in DMF afforded 1-(2-phenoxyethyl)-4-(pyridine-2-yl)piperazines 42 in 45–59% yields (Scheme 14).

**Scheme 15:**
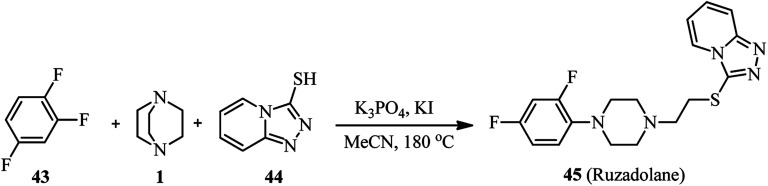
Synthesis of ruzadolane *via* DABCO bond cleavage strategy.

This protocol was successfully applied for the synthesis of analgesic ruzadolane 45 (Scheme 15). For this purpose, the one-pot three-component reaction of 1,2,4-trifuorobenzene 43, DABCO 1, and [1,2,4]triazolo[4,3-*a*]pyridine-3-thiol 44 in the presence of K_3_PO_4_ and KI was carried out to provide the desired drug in 55% yield at 180 °C for 72 h, or 52% yield under microwave heating for 20 h.

**Scheme 16:**
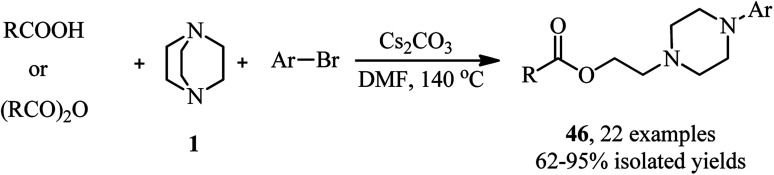
Carboxylic acids and anhydrides as nucleophile in DABCO bond cleavage.

Recently, the same group reported a novel one-pot three component reaction for the synthesis of aromatic aminoalkyl esters 46*via* a direct C–N esterification/arylation reaction. For this purpose, reactions of an activated aryl halide (4-bromonitrobenzene) or a variety of pyridyl halides with carboxylic acids and DABCO were carried out in the presence of 100 mol% of Cs_2_CO_3_ in DMF at 140C for 12 h (Scheme 16).^35^ The products were obtained in high to excellent yields. In addition, they have shown that acid anhydrides can be used instead of carboxylic acids in this protocol under similar conditions. They also confirmed that the reactivity of carboxylic acids and anhydrides was not affected by the steric hindrance of substituents. In addition, *N*-methylpyrrolidine was applied successfully in this protocol instead of DABCO to afford the corresponding linear aminoesters in high yields.

**Scheme 17:**
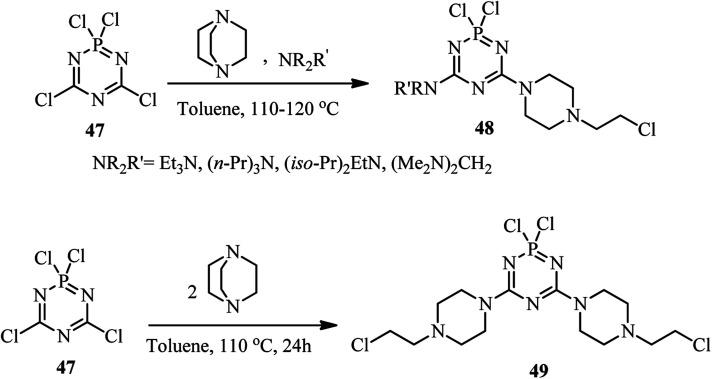
Synthesis of 4-(2-chloroethyl)piperazino derivatives of carbaphosphazenes.

The activation of DABCO by novel heteroarenes is further investigated using carbaphosphazenes. In this case, Reddy *et al.* developed a new method for preparing 4-(2-chloroethyl)piperazino derivatives of carbaphosphazenes 48 and 49*via* the reaction of DABCO and tetrachlorocyclodicarbaphosphatriazene 47 (Scheme 17).^36^ By using an equimolar amount of 47, 1, and an acyclic tertiary amine, the corresponding piperazino heterocycles 48 were obtained in 32–82% isolated yields. Beside the DABCO, a range of tertiary amines was utilized as a third component in 1 : 1 : 1 molar ratio to increase the diversity of the reaction and solubility of the products. In addition, quinuclidine was applied successfully instead of DABCO in this protocol. Other cyclic tertiary amines such as *N*-methylmorpholine and *N*-methylpiperidine did not show any evidence of ring cleaved products. They also have shown that the reaction of 47 with DABCO in 1 : 2 molar ratio afforded the bis piperazino product 49 in 56% yield *via* two DABCO ring opening reactions by chloride attack. The cleaved alkyl group of DABCO remains as an alkyl chloride on the product molecule.

**Scheme 18:**
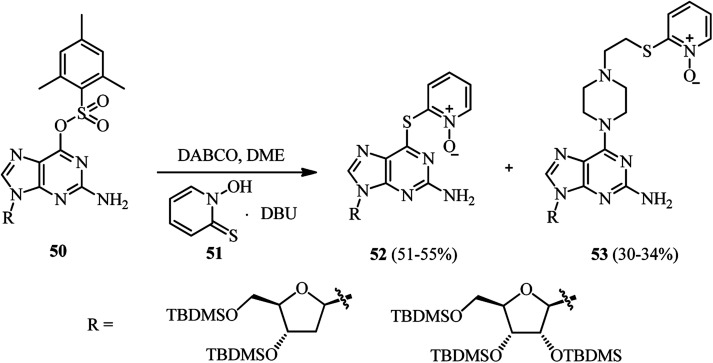
Synthesis of piperazine substituted nucleoside *via* DABCO bond cleavage.

Finally, during the introducing of *N*-hydroxypyridine-2(1*H*)-thione (NHPT) 51 at the 6-position of 2′-deoxyguanosine 50 in the presence of DABCO, Vrantza *et al.* observed that the corresponding piperazine substituted nucleoside 53 were obtained as side products in 30–34% yields (Scheme 18).^37^ In fact, DABCO has dual role as catalyst and reactant in this protocol. The reaction proceeded *via* DABCO attack to the position 6 of the nucleoside 50, then S-nucleophilic attack of NHPT at the α-C-atom of the positively charged DABCO–purine intermediate, followed by an S_N^2^_ addition and opening of the ethylene DABCO bridge.

## 4. Benzynes as activating agents

**Scheme 19:**
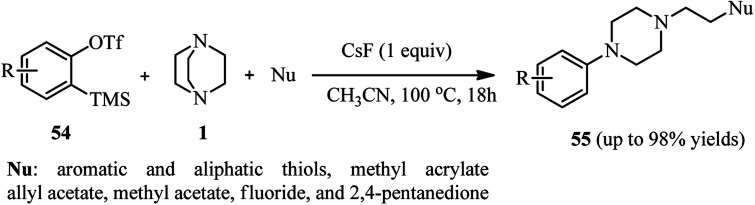
DABCO bond cleavage by *in situ* prepared benzyne and a nucleophile.

Benzynes are momentous intermediates for the preparation of significant scaffold in chemistry. Using benzynes as activator for DABCO bond cleavage is developed by Min *et al.*^38^ For this purpose, a one-pot three-component reaction of an equivalent of a benzyne precursor 54 [2-(trimethylsilyl)phenyl trifluoromethanesulfonate or its derivatives], two equivalents of DABCO 1, and an excess amount of a nucleophile in the present of an equivalent of CsF in CH_3_CN at 100 °C for 18 h was carried out to afford the 2-(4-arylpiperazin-1-yl)ethyl-containing molecules 55 in 18–98% isolated yields. Beside thiols, other nucleophiles such as methyl acrylate, allyl acetate, methyl acetate, fluoride, and 2,4-pentanedione participated well in the DABCO ring opening reaction to form C–O, C–C, and C–F bonds (Scheme 19).

**Scheme 20:**
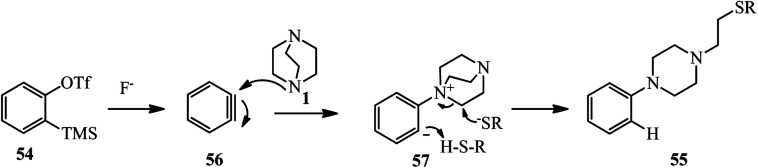
Proposed mechanism for DABCO bond cleavage by *in situ* prepared benzyne.

The mechanism of this reaction is proposed in Scheme 20. The benzyne 56 generated *in situ* from *o*-silyl aryl triflates 54 and CsF is attacked by DABCO 1 to generate zwitterion intermediate ammonium salt 57. The intermediate 57 undergoes ring opening with the aid of a nucleophile to furnish 1-ethyl-4-arylpiperazine derivative 55.

**Scheme 21:**
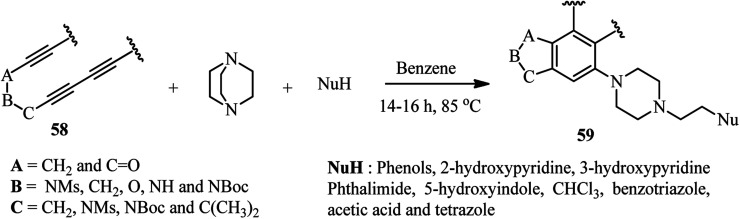
One-pot three-component reaction of polyynes, DABCO, and a protic nucleophile.

Scheme 21 depicts a facile and highly efficient strategy for the construction of multiheterocyclic compounds which was reported by Ross and Hoye in 2018.^39^ They have shown that the three-component reaction of a polyyne 58, DABCO, and a protic nucleophile (NuH) afforded the corresponding multiheterocyclic products 59 in good to high yields (36–74%). Various tri- and tetraynes were applied successfully to construct diversities of fused bicyclic compounds. In addition, various protic nucleophiles such as phenols with electron-donating and withdrawing groups, hydroxypyridines, 5-hydroxyindole, phthalimide, acetic acid, tetrazole and benzotriazole were introduced in the structure of products. By performing the reaction in chloroform solution, they have shown that chloride (from chloroform) can be introduced in the structure of product and act as nucleophile in the DABCO ring opening step.

**Scheme 22:**
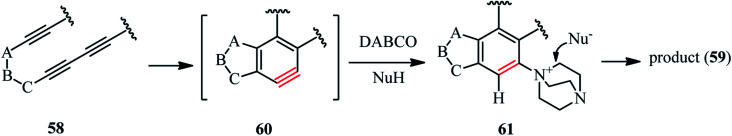
Proposed mechanism for the synthesis of multiheterocyclic products from polyynes, DABCO and nucleophiles.

They proposed that the thermal cycloisomerization of tethered tri- and tetraynes 58 leads to benzynes 60 under neutral conditions in a hexadehydro-Diels–Alder reaction. In the presence of DABCO and a proton source, the benzyne intermediate 60 converted to ion pair 61, followed by DABCO ring opening to afford the products (Scheme 22). The following criteria should be considered for this type of reaction: (i) neither the DABCO or the protic nucleophile should not react with the polyyne precursor faster than its rate of cyclization; (ii) the DABCO should add to the benzyne intermediate faster than the protic nucleophile; (iii) the protic nucleophile should be acidic enough to protonate the intermediate 1,3-zwitterion; and (iv) the conjugate base of H-Nu should be sufficiently nucleophilic to undergo DABCO ring opening. By considering these limitations, a one-pot two-step strategy involving the formation and subsequent nucleophilic ring-opening of the intermediate 61 is recommended.

**Scheme 23:**
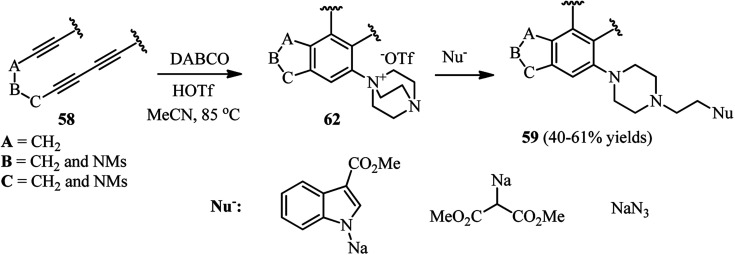
Reaction of polyynes with DABCO in the present of trifilic acid and subsequent ring opening with a nucleophile.

In addition, they have shown that by using HOSO_2_CF_3_ as protic nucleophile in the same reaction, the corresponding DABCO ammonium triflate intermediates 62 can be obtained, which is suitable intermediate for ring opening by various nucleophiles including sodium salts of methyl indole-3-carboxylate, dimethylmalonate, and azide (Scheme 23).

**Scheme 24:**
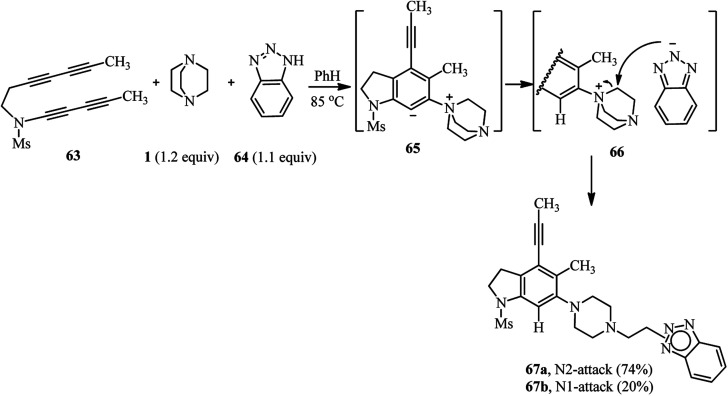
One-pot three component reaction of tetrayne, DABCO and benzotriazole.

**Scheme 25:**
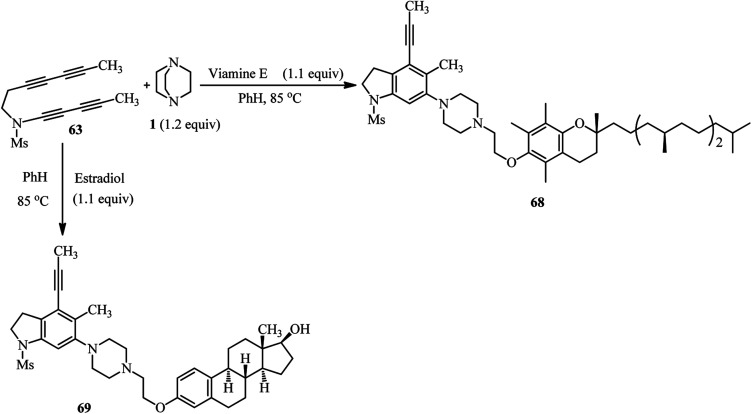
Utilization of phenolic natural products as nucleophiles in benzyne-activated DABCO bond cleavage.

The same authors have shown that trapping of the *in situ* prepared benzyne from tetrayne 63 with DABCO in the presence of benzotriazole 64 furnish the 1,3-zwitterion 65, and then the ammonium-benzotriazolide ion pair 66, which finally undergoes nucleophilic ring opening through the N^1^- or N^2^-position of benzotriazolide to afford the products 67a (74%) and 67b (20%), respectively (Scheme 24).^40^ This approach was successfully applied for the functionalization of phenolic natural products such as vitamin E and estradiol. By heating tetrayne (1 equiv.), DABCO (1.2 equiv.), and vitamin E (1.1 equiv.) (Scheme 25, right) or estradiol (1.1 equiv.) (Scheme 25, down) in benzene at 85 °C, the corresponding products 68 and 69 were obtained in 64% and 79%, respectively. Although estradiol contains both alcoholic and phenolic groups in the structure as potential nucleophiles, the reaction proceeded chemoselectively *via* the phenolic group.

## 5. Transition-metal catalyzed DABCO bond cleavage

**Scheme 26:**
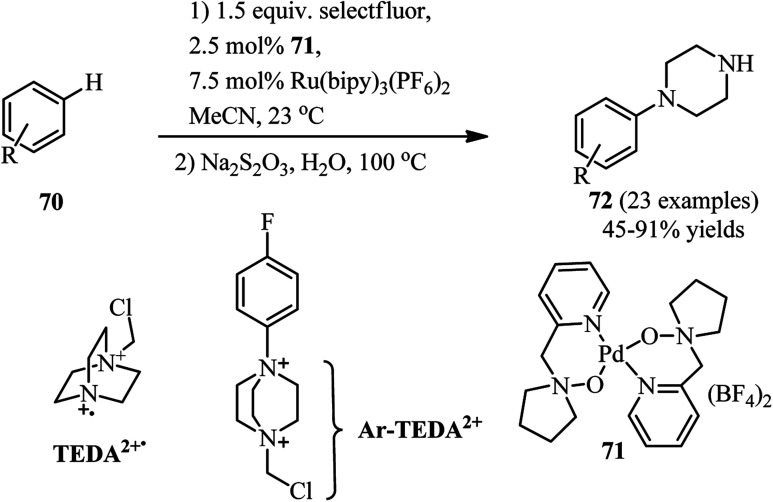
Transition-metal catalyzed piperazination of arenes.

Transition-metal catalyzed C–H activation in aryl rings or C–X activation in aryl and vinyl halides is an efficient strategy for the synthesis of DABCO salts suitable for C–N bond cleavage. For this purpose, a one-pot two-step procedure for the regioselective synthesis of aryl piperazines 72 from the corresponding C–H compounds and Selectfluor® as DABCO derivative is developed by Boursalian *et al.* under irradiation-free conditions (Scheme 26).^41^ This reaction was catalyzed by a dual catalyst combination: a palladium complex 71 (2.5 mol%) and Ru(bipy)_3_(PF_6_)_2_ (7.5 mol%). They hypothesized that a single-electron reduction of Selectfluor® provided the corresponding doubly cationic radical TEDA^2+^˙ (TEDA, *N*-(chloromethyl)triethylenediamine), which is capable to undergo radical aromatic substitution to give *N*-aryl-*N*′-chloromethyldiazoniabicyclo[2.2.2]octane salts (Ar-TEDA^2+^). The reaction was continued by reduction of Ar-TEDA^2+^ compounds by sodium thiosulfate. Various arenes including five- and six-membered (hetero)arenes undergo piperazination in this protocol. The site of piperazination can be determined by directing group on the aromatic system.

**Scheme 27:**
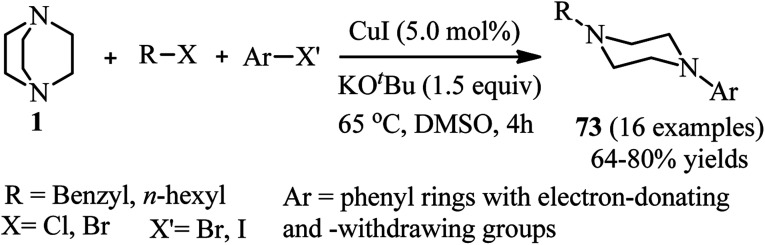
Cu(i)-catalyzed synthesis of *N*-alkyl-*N*′-aryl-piperazines.

Cu(i)-catalyzed synthesis of *N*-alkyl-*N*′-aryl-piperazines 73*via* a one-pot three-component reaction of DABCO, alkyl halides, and aryl halides is reported by Yavari *et al.* in 2014. The reactions were carried out in the presence of a catalytic amount of copper iodide (5 mol%) and KO*t*Bu as base in DMSO at 65 °C. Alkyl chlorides and bromides and aryl bromides and iodides were applied successfully in this protocol to afford the unsymmetrical piperazines in high to excellent yields (Scheme 27).^42^

**Scheme 28:**
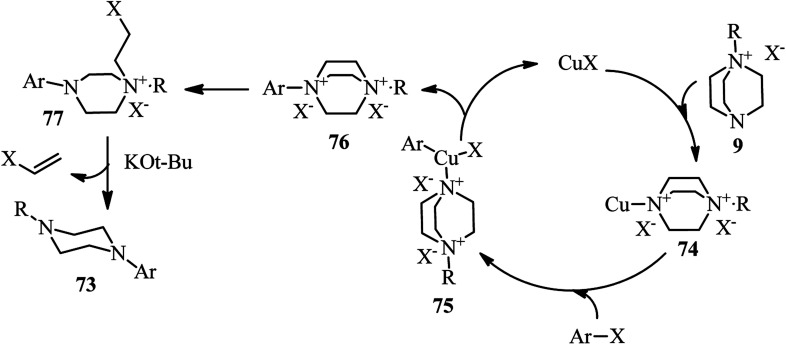
Proposed mechanism for Cu(i)-catalyzed synthesis of *N*-alkyl-*N*′-aryl-piperazines.

The proposed mechanism by authors is depicted in Scheme 28. They proposed that the crucial step is the formation of DABCO salt 9. This salt then coordinated to CuI *via* free nitrogen atom to afford the intermediate 74. Then, aryl halide could be oxidatively added to the Cu(i)-complex 74 to form Cu(iii)-intermediate 75. Reductive elimination of the intermediate 75 could provide the ammonium salt 76. This intermediate undergoes nucleophilic displacement by chloride ion to afford the intermediate 77, followed by Hofmann elimination in the presence of a base to furnish the final product 73. The proposed mechanism is supported by the fact that iodoethylene can be detected in GC-MS analysis.

**Scheme 29:**
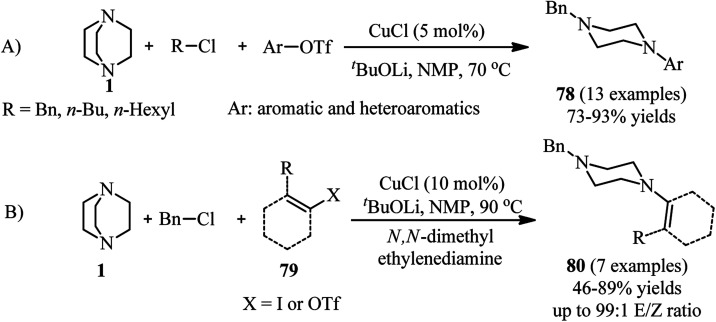
Cu(i)-catalyzed synthesis of piperazines using aryl triflates or alkenyl iodides (triflates).

In addition, the synthesis of unsymmetrical piperazines with the same strategy is further developed by Ghazanfarpour-Darjani and coworkers.^43^ They have utilized alkyl chlorides, aryl(heteroaryl) triflates and DABCO under optimal reaction conditions [CuCl (5 mol%), *t*-BuOLi (1.5 equiv.), and NMP as solvent at 70 °C for 14 h] to provide *N*-alkyl-*N*′-aryl (heteroaryl) piperazines 78 in 73–93% yields. They also concluded that by using alkenyl triflates 79 instead of aryl triflates or aryl iodides, the corresponding *N*-alkyl-*N*′-alkenyl piperazines 80 can be prepared in 46–89% yields. The use of *N*,*N*′-dimethyl ethylenediamine as ligand and performing the reactions at higher temperature (90 °C) is crucial for this reaction. Both external and internal alkenyl iodides (triflates) are suitable substrates in this protocol (Scheme 29).

## 6. Pyridine-*N*-oxides as activating agents

**Scheme 30:**
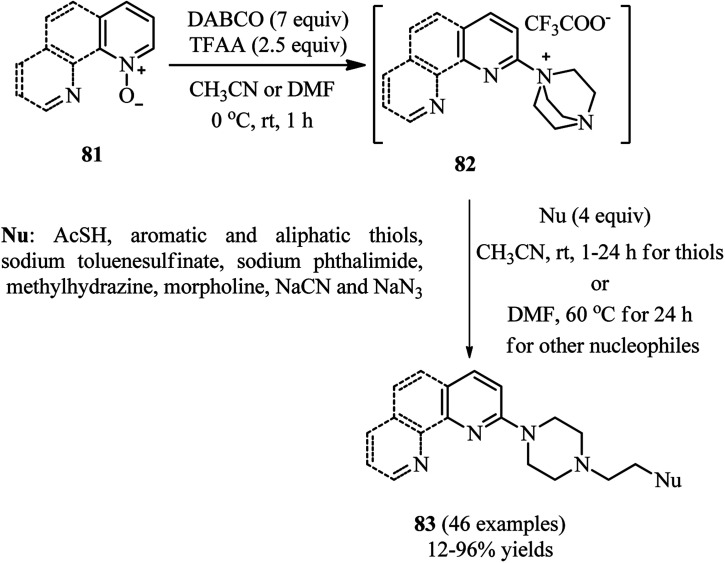
DABCO bond cleavage using pyridine-*N*-oxides, TFAA and a nucleophile.

**Scheme 31:**
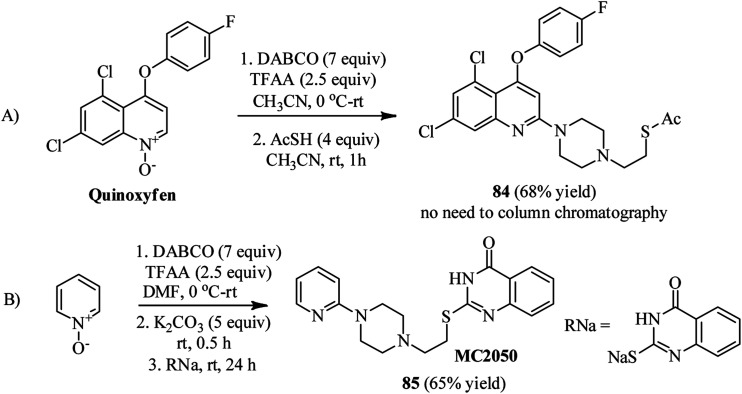
Synthesis of functionalized Quinoxyfen–piperazine and MC2050 starting from pyridine-*N*-oxides.

Bugaenko *et al.* reported that the quaternary *N*-(2-pyridyl)-DABCO salts 82 can be simply prepared by the reaction of heterocyclic *N*-oxides 81 with DABCO in the presence of an activating agent such as TFAA at 0 °C to rt for 1 h. This intermediate 82 can be simply react with diversities of nucleophiles such as thioacetic acid, aromatic and aliphatic thiols, phthalimide, methylhydrazine, morpholine, sodium cyanide, sodium benzenesulfinate to give the functionalized *N*-(2-pyridyl)-*N*′-ethylpiperazines 83 in moderate to excellent yields (12–96%) *via* DABCO ring opening reaction (Scheme 30).^44^ Diversely substituted pyridine *N*-oxides, quinolone-*N*-oxides, and 1,10-phenanthroline 1-oxide were applied successfully in this protocol. In addition, they have confirmed that many nucleophile-sensitive and synthetically valuable functional groups including halide, ester, amide, and nitrile were perfectly tolerated in this protocol. The main advantages of this protocol including operationally simple, metal-free, one-pot synthetic procedure, mild reaction conditions, high positional selectivity, and excellent functional group tolerance are noteworthy. The potential of this protocol for the synthesis of drug-like molecules 84–85 were examined for the late stage functionalization of Quinoxyfen and for one-pot synthesis of MC2050, a potent PARP-1 inhibitor, in reasonable isolated yield 65% *versus* 45% in two-step process (Scheme 31).^45^

**Scheme 32:**
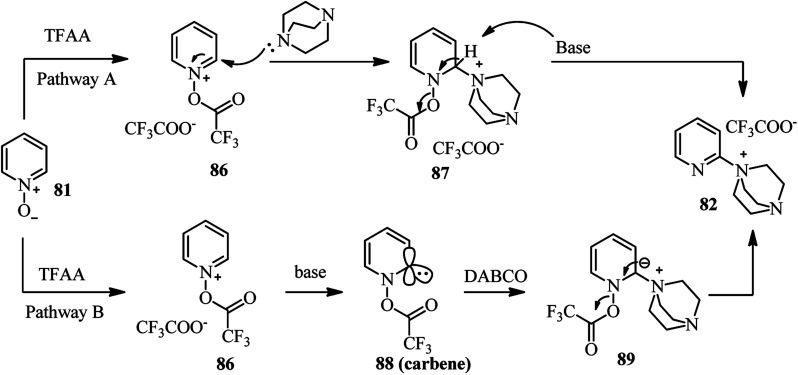
Proposed mechanisms for the synthesis of *N*-(2-pyridyl)-DABCO salt from pyridine-*N*-oxide.

Two reaction pathways for the preparation of the intermediate 82 are recommended by authors (Scheme 32). In both cases, activation of *N*-oxide by TFAA was considered as initial event which enhance both electrophilicity and CH-acidity of the C-2 position in pyridine ring. Then, DABCO attack to the C-2 position of 86, followed by deprotonation/aromatization provides the intermediate 82 (Scheme 32, pathway A). Alternatively, abstraction of the acidic hydrogen in position C-2 of the pyridine ring in 86 by DABCO can provide carbene 88 which is active electrophilic specie toward nucleophilic attack by DABCO. Finally, aromatization provides the salt 82 (Scheme 32, pathway B).

**Scheme 33:**
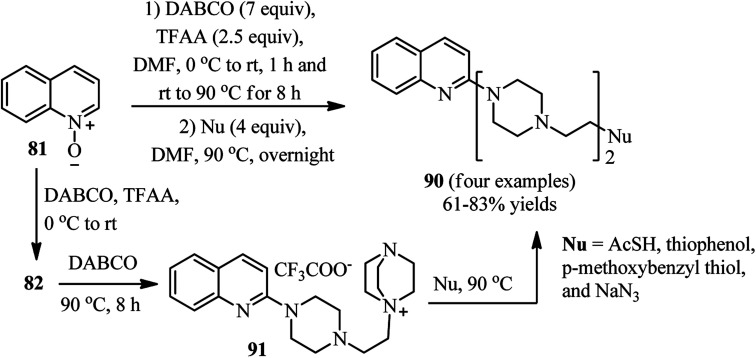
Synthesis of quinolones containing bis(ethylpiperazine) motif.

The same group also described that by varying the reaction conditions including raising the reaction temperature and time in both steps of the reaction (0 °C rt for 1 h and rt-90 °C for 8 h for the first step; 90 °C for overnight for the second step), it is possible to prepared the potential biologically active heterocyclic compounds 90 comprising bis(ethylpiperazine) motif. They hypothesized that the ammonium salt 91 acts as intermediate in this protocol and is reactive for further ring opening (Scheme 33).^44^

**Scheme 34:**
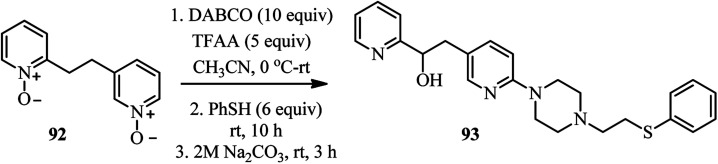
Using bis-*N*-oxide in DABCO bond cleavage reaction.

Interestingly, they have also shown that by using bis-*N*-oxide 92 containing two alkylated pyridine moieties in the standard protocol described in the Scheme 30, the corresponding complex heterocyclic compound 93 was obtained in 76% yield and complete regioselectivity (Scheme 34). They described that while the 3-alkylpyridine part reacted in the typical manner to produce a substituted piperazine, the 2-alkylpyridine fragment underwent the Boekelheide reaction, to afford the hydroxyl group after hydrolysis.^44^

## 7. Diaryliodonium triflates as activating agents

**Scheme 35:**
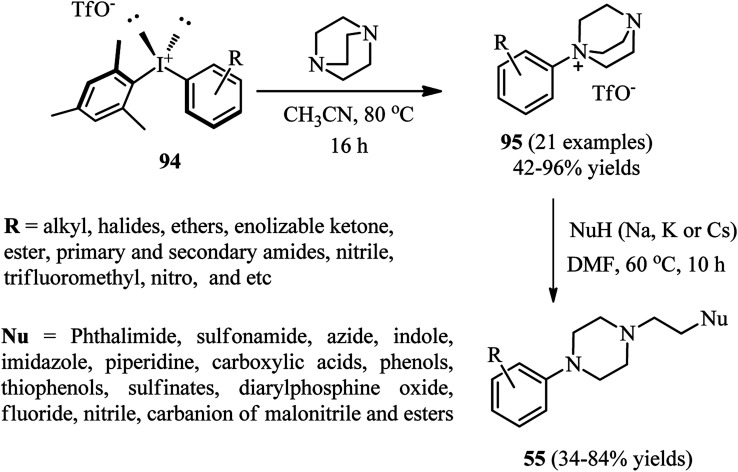
Aryl(mesityl)iodonium triflates as activating agents for DABCO bond cleavage.

**Scheme 36:**
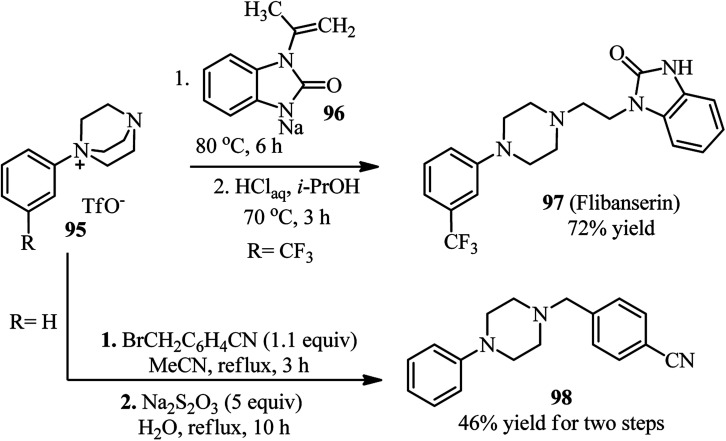
Synthesis of biologically active compounds from *N*-aryl-DABCO salts.

Recently, Karchava *et al.* introduced the diaryliodonium salts 94 as efficient arylating agents for a tertiary sp^3^-nitrogen.^46^ For this purpose, reaction of DABCO 1 (1 equiv.) with various aryl(mesityl)iodonium triflates 94 (2 equiv.) was carried out in acetonitrile at 80 °C for 16 h to prepare the corresponding *N*-aryl-DABCO salts 95 with both electron-donating and electron-withdrawing groups in different positions of the aryl substituent in 42–96% isolated yields (Scheme 35). In continue, the reaction of salts 95 with various nucleophiles NuH (K, Na, Cs) including phthalimide, solfonamide, azide, indole-3-carbaldehyde, imidazole, 4-benzylpiperidine, carboxylic acid salts, phenols, thiophenols, diphenylphosphine oxide, sodium benzenesulfinate, fluoride, cyanide, and malonitrile were performed in DMF at 60 °C for 10 h to afford the functionalized piperazines 55 as drug-like scaffolds, ligands, and synthetically useful compounds (Scheme 35). The synthetic usefulness of this protocol was examined for the synthesis of Flibanserin 97, the active agent of recently approved drug Addyi, in three steps starting from commercially available 1-isopropenylbenzimidazol-2-one 96 and salt 95 (R = CF_3_). In addition, they have shown that the reaction of salt 95 with an alkylating agent like *p*-cyanobenzyl bromide, followed by treatment with Na_2_S_2_O_3_ afforded the corresponding piperazines 98 in good yield (Scheme 36).

**Scheme 37:**
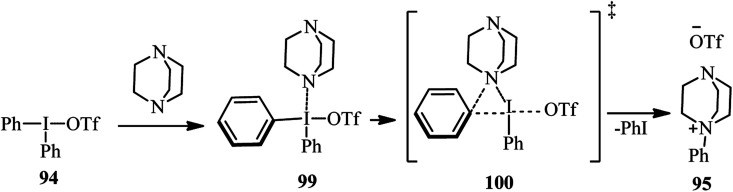
Proposed mechanism for the reaction of DABCO with diaryliodonium triflate.

The authors proposed that the reaction of DABCO with diaryliodonium triflate 94 proceeds *via* an initial formation of 99, followed by a concerted ligand coupling at the iodine center to provide the salt 95. The structure of intermediate 99 was confirmed by X-ray analysis, in which the iodine center adapts a tetracoordinated planar arrangement with an N⋯I bond length of 2.848 Å (Scheme 37).

## 8. *In situ* prepared nitrile as activating agent

**Scheme 38:**
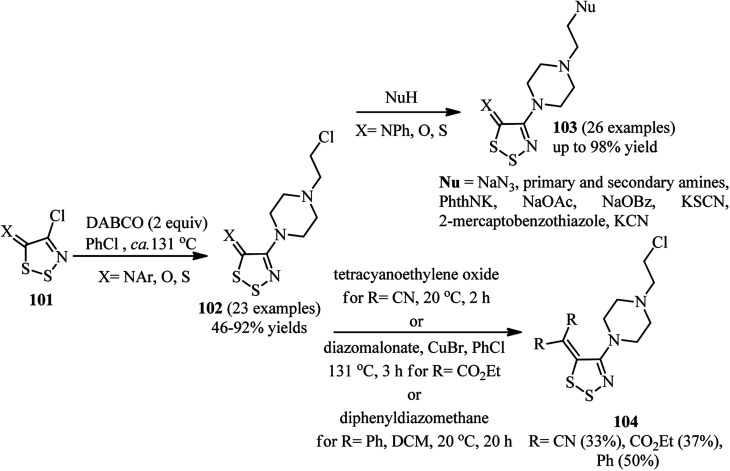
Introducing 4-(2-chloroethyl)piperazinyl group on the C4 position of 1,2,3-dithiazoles and their postfuncionalization.

A general method for introducing 4-(2-chloroethyl)piperazinyl group on the C4 position of 1,2,3-dithiazoles is developed by Koyioni *et al.*^47^ They concluded that reaction of *N*-(4-chloro-5*H*-1,2,3-dithiazol-5-ylidene)-anilines or 4-chloro-5*H*-1,2,3-dithiazol-5-one (thione) 101 with DABCO in hot PhCl at 131 °C afforded the corresponding products 102 in high to excellent yields (Scheme 38). After successful synthesis of 102, their reaction with various nucleophiles such as NaN_3_, primary and secondary amines, potassium phthalimide, sodium acetate, sodium benzoate, potassium thiocyanate, thiols and potassium cyanide were carried out to give the appropriate products 103 with up to 97% isolated yields. While no reaction was occurred between DABCO and various (4-chloro-5*H*-1,2,3-dithiazol-5-ylidene)methanes, the corresponding products 104 were obtained *via* the C5 postfunctionalization of the 4-(2-chloroethyl)piperazinyl dithiazolethione 102 in modest yields.

**Scheme 39:**
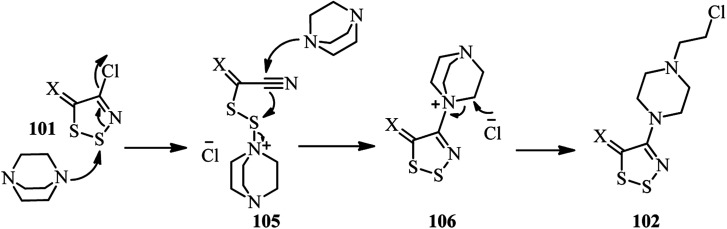
Proposed mechanism for the reaction of 101 with DABCO.

The authors proposed that the reaction proceeded *via* the ring-opening of the 1,2,3-dithiazole 101 by nucleophilic attack of DABCO on the sulfur S2 to afford the disulfide intermediate 105. Nucleophilic attack of the second molecule of DABCO to the nitrile group provided an amidine which then cyclizes onto the disulfide 106 to release the initial DABCO. Finally, the DABCO ring opening was occurred by chloride attack. In summary, the reaction proceeded *via* addition of nucleophile, ring-opening, and ring closure mechanism to prepared the corresponding DABCO salt (Scheme 39).

## 9. Alkynes as activating agent

**Scheme 40:**
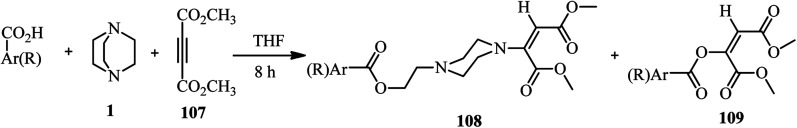
One-pot three-component reaction of DABCO, DMAD and carboxylic acids.

Using alkynes as activator for DABCO ring opening is introduced by Dong and coworkers. They investigated the reaction of DABCO, a carboxylic acid, and an activated alkyne such as dimethyl acetylenedicarboxylate (DMAD) 107 under catalyst-free conditions in THF (Scheme 40).^48^ Various aromatic and aliphatic carboxylic acids were tolerated in this protocol. Not only DMAD, but also alkyl propiolates were examined as suitable substrates in this protocol. The role of DABCO is important in this protocol; while in the present of a catalytic amount of DABCO the main product is Michael addition of carboxylic acids to the alkynes, by using a stoichiometric amount of DABCO the corresponding piperazine adducts 108 were obtained in moderate to excellent yields. The configuration of the molecule around double bond was examines as *E* by X-ray crystallography.

**Scheme 41:**
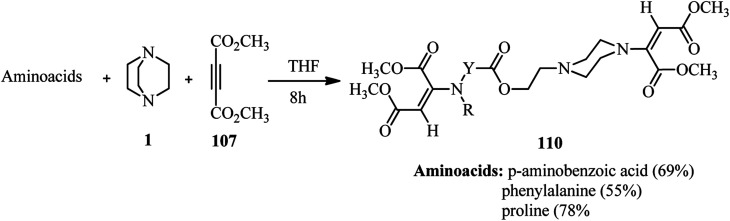
One-pot pseudo three-component reaction of DABCO, DMAD and amino acids.

This protocol was applied for the synthesis of various (un)substituted amino carboxylic acid piperazine derivatives 110 by using amino acids instead of carboxylic acids. In the presence of amino acids, the products with bis(*E*)-1,2-(dimethoxycarbonyl)ethen-1-yl moiety were obtained in high yields (Scheme 41).

## 10. Sulfonyl chlorides as activating agents

**Scheme 42:**
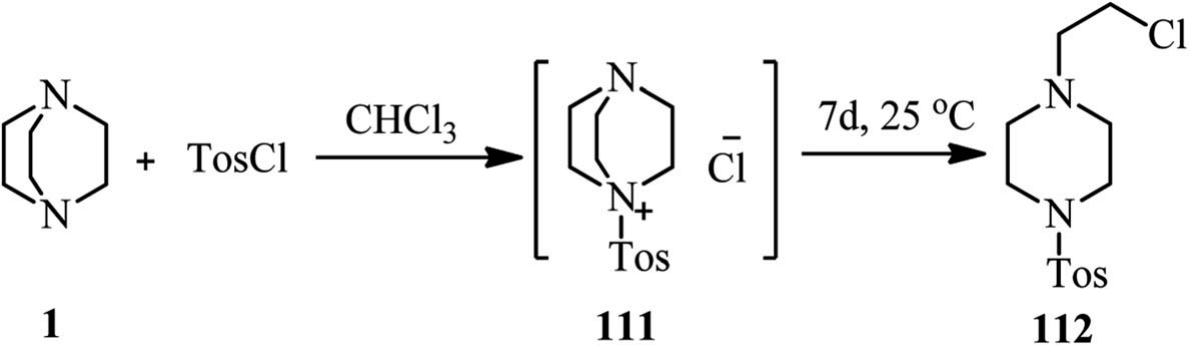
Reaction of DABCO with tosyl chloride.

Although it is well documented that DABCO is an efficient substitute for pyridine in tosylation reactions and for triethylamine in sulfenylation reactions, but Hartung *et al.* have shown that by prolonging the reaction time in these reactions, the corresponding sulfonamide 112 can be isolated as side product *via* DABCO bond cleavage (Scheme 42).^49^

**Scheme 43:**
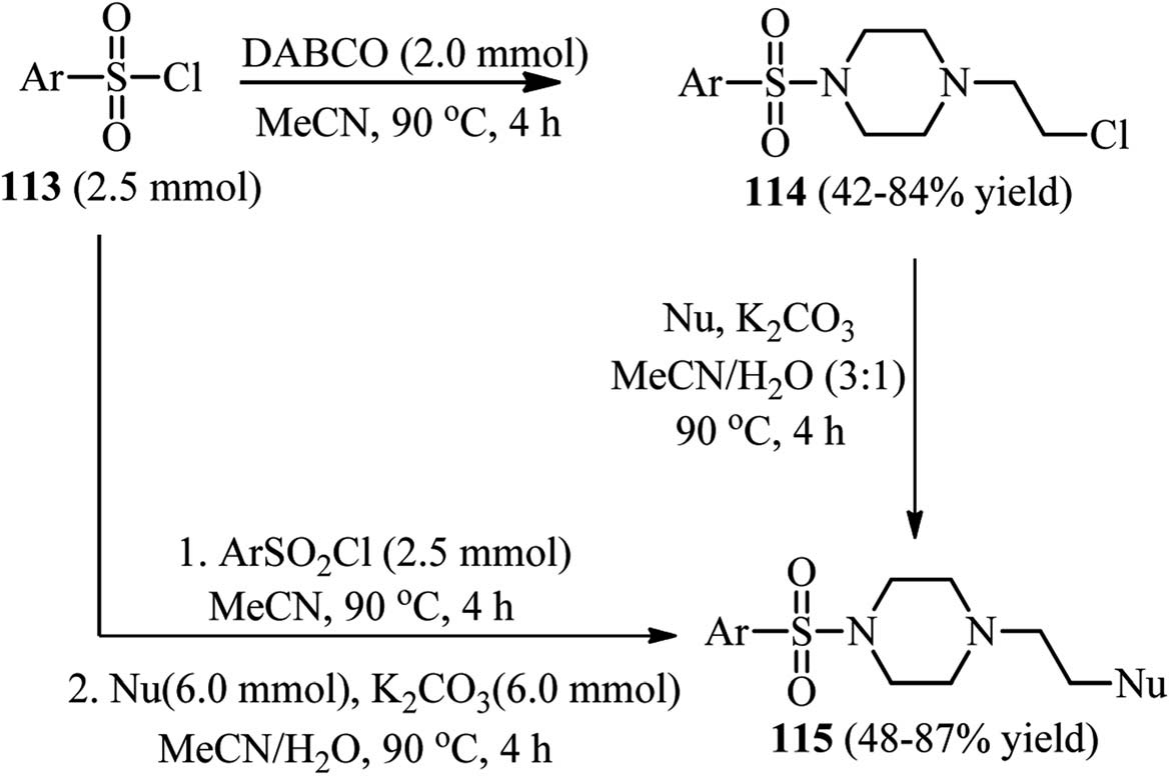
A one-pot or two-pot strategy for the synthesis of 1-(2-substitued ethyl)-4-sulfonylpiperazines.

This strategy has been further developed by Fu *et al.* for the synthesis of 1-(2-substitued ethyl)-4-sulfonylpiperazines 115.^50^ They concluded that these compounds can be simply prepared *via* a two-step protocol by separation of the 1-(2-chloroethyl)-4-sulfonylpiperazine intermediate 114 and then reaction with a nucleophile or *via* a one-pot two-step reaction of DABCO with a sulfonyl chloride, followed by addition of a nucleophile (Scheme 43). While aryl(heteroaryl) sulfonyl chlorides gave moderate to high yields, aliphatic sulfonyl chlorides do not participate in this protocol. Various nucleophiles including thiols, sodium methoxide, potassium thioacetate, potassium thiocyanate, secondary aromatic amines, carboxylic acids, phenols, and sodium benzene sulfinate were applied successfully in this protocol. According to the spectroscopic studies, the authors confirmed that the charge-transfer complex formation between sulfonyl chlorides and DABCO facilitates the C–N bond cleavage of DABCO.

## 11. Miscellaneous

**Scheme 44:**
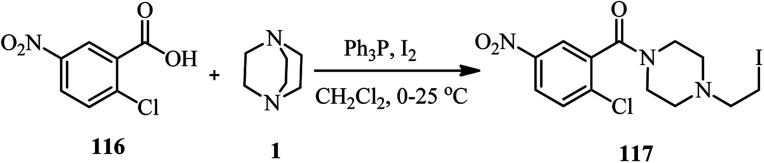
Synthesis of piperazines *via* direct amidation of carboxylic acid with DABCO.

A metal-free direct amidation of carboxylic acid 116 with tertiary amines in the presence of Ph_3_P–I_2_ is reported by Phakhodee *et al.* in 2016. They have shown that when DABCO was applied as a nitrogen source in the reaction with an acid, the DABCO ring underwent endocyclic C–N bond cleavage to afford the iodo-substituted amide product 117 in 52% yield (Scheme 44).^51^

**Scheme 45:**
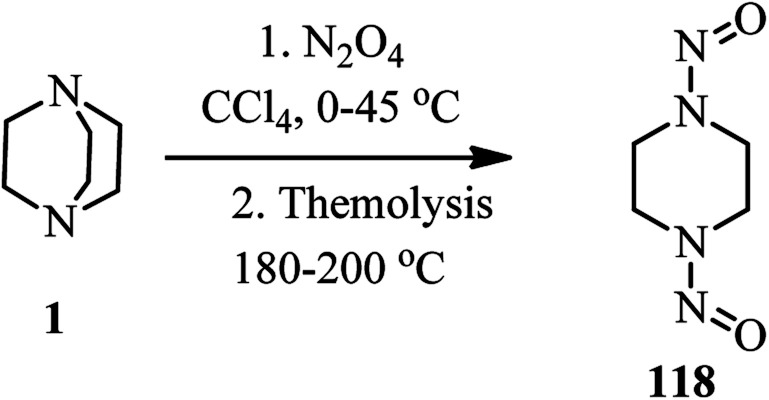
Synthesis of 1,4-dinitrosopiperazine from DABCO and N_2_O_4._

Reaction of DABCO with an excess amount of dinitrogen tetroxide in CCl_4_ at 0–40 °C, followed by recrystalisation in ethanol and nitric acid afforded the corresponding diamine dinitrate in 74% isolated yield. Thermolysis of the diamine dinitrate salt at 180–200 °C gave the 1,4-dinitrosopiperazine 118 in low yield (Scheme 45).^52^

**Scheme 46:**
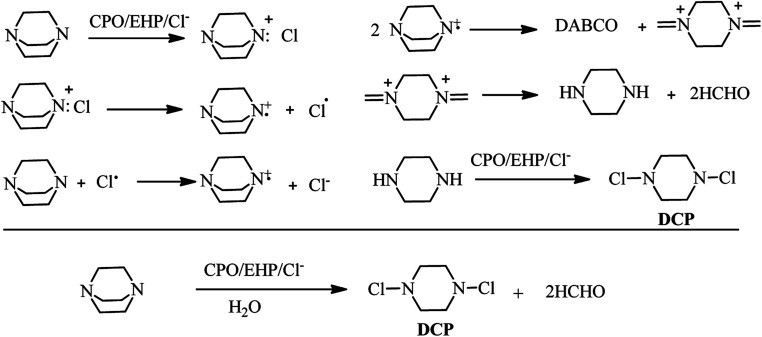
Enzyme-mediated oxidation of DABCO by ethylhydroperoxide (EHP).

Enzyme-mediated oxidation of DABCO by H_2_O_2_ or ethylhydroperoxide (EHP) in the presence of chloride Cl^−^ was published by Sayo *et al.* in 1988. They concluded that reaction of DABCO with chloroperoxidase (CPO)–EHP–Cl system at pH = 5 afforded the piperazine, dichloropiperazine (DCP), and formaldehyde as products. According to ESR experiments, they identified that the reaction proceeded *via* formation of DABCO chloroammonium cation, followed by homolysis of the cation (Scheme 46).^53^

**Scheme 47:**
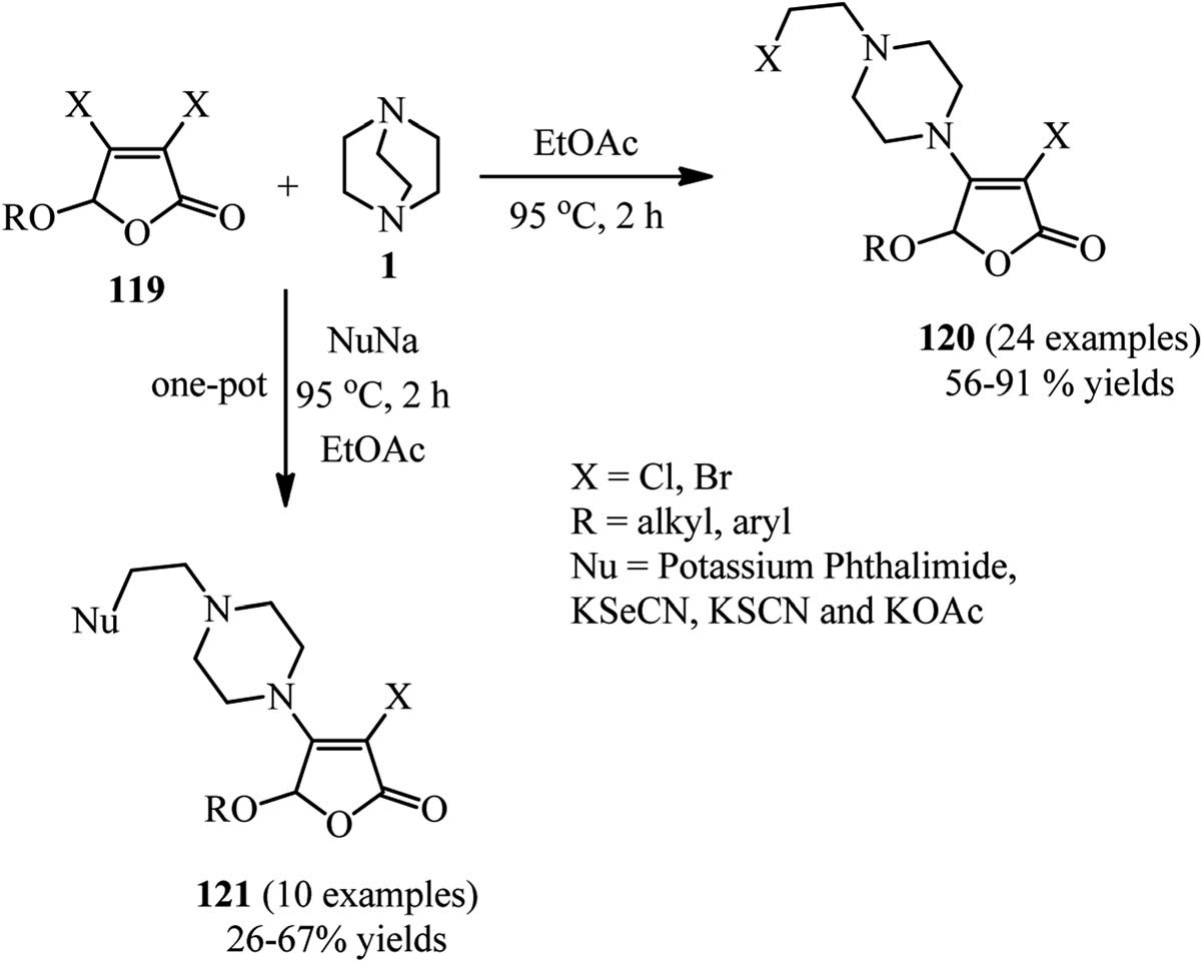
Synthesis of piperazines using 3,4-dihalo-2(5*H*)-furanone.

Finally, a green and environmental benign procedure for the synthesis of functionalized 4-ethylpiperazines *via* a 3,4-dihalo-2(5*H*)-furanone 119 initiated DABCO C–N bond cleavage under catalyst-free conditions is reported by Wu *et al.*^54^ They have shown that reaction of DABCO with a 3,4-dihalo-2(5*H*)-furanone 119 in ethyl acetate at 95 °C under air atmosphere for 2 h afforded the corresponding furanone derivatives containing piperazine motif on the 4-position 120 in 56–91% yield with 100% atom economy (Scheme 47). Both 3,4-dibromo-2(5*H*)-furanone and 3,4-dichloro-2(5*H*)-furanone are good substrates in this protocol. After successful synthesis of chloroethylpiperazine derivatives, displacement of the chloride in 120 with a nucleophile was carried out *via* a one-pot three-component reaction of DABCO, 119, and a nucleophile such as potassium salts of phthalimide, selenocyanate, and thiocyanate. The corresponding products 121 were obtained in 26–67% isolated yields.

## 12. Conclusion

Synthesis of functionalized piperazine derivatives *via* C–N bond cleavage can be conveniently achieved using DABCO as the key substrate. Activation of DABCO with various reagents such as alkyl halides, aryl(heteroary) halides, carboxylic acids, diaryliodonium salts, tosyl halides, activated alkynes, benzynes and *etc.* provided the corresponding quaternary ammonium salts of DABCO, which are very good electrophiles for various nucleophiles for C–O, C–S, C–N, C–P and C–C bond formations. Besides pre-activated DABCO salts, the *in situ* activation of DABCO in multicomponent reactions were also applied for efficient synthesis of piperazine derivatives. The importance of functionalized piperazine derivatives in the development of novel drugs and biologically active compounds inspired the organic chemists to employ DABCO bond cleavage technique as a simple and efficient protocol in synthetic organic chemistry for the diversity oriented synthesis of these types of compounds. In this scenario, the dominance of DABCO bond cleavage technique in piperazines synthesis will definitely continue as long as our quest for novel functionalized piperazine derivatives continues.

## Conflicts of interest

There are no conflicts to declare.

## Supplementary Material

## References

[cit1] Gomtsyan A. (2012). Chem. Heterocycl. Compd..

[cit2] Baumann M., Baxendale I. R. (2013). Beilstein J. Org. Chem..

[cit3] Cabrele C., Reiser O. (2016). J. Org. Chem..

[cit4] Vitaku E., Smith D. T., Njardarson J. T. (2014). J. Med. Chem..

[cit5] Taylor R. D., MacCoss M., Lawson A. D. G. (2014). J. Med. Chem..

[cit6] Thanban Chandrika N., Shrestha S. K., Ngo H. X., Tsodikov O. V., Howard K. C., Garneau-Tsodikova S. (2018). J. Med. Chem..

[cit7] Pytka K., Rapacz A., Zygmunt M., Olczyk A., Waszkielewicz A., Sapa J., Filipek B. (2015). Pharmacol. Rep..

[cit8] Parai M. K., Panda G., Srivastava K., Puri S. K. (2008). Bioorg. Med. Chem. Lett..

[cit9] Brown A. M., Patch T. L., Kaumann A. J. (1991). Br. J. Pharmacol..

[cit10] Le Bihan G., Rondu F., Pele-Tounian A., Wang X., Lidy S., Touboul E., Lamouri A., Dive G., Huet J., Pfeiffer B., Renard P., Guardiola-Lemaitre B., Manechez D., Penicaud L., Ktorza A., Godfroid J. J. (1999). J. Med. Chem..

[cit11] Ranise A., Spallarossa A., Bruno O., Schenone S., Fossa P., Menozzi G., Bondavalli F., Mosti L., Capuano A., Mazzeo F., Falcone G., Filippelli W. (2003). Farmaco.

[cit12] McNair M. D. T. J., Wibin M. D. F. A., Hoppe E. T., Schmidt M. D. J. L., dePeyster M. D. F. A. (1963). J. Surg. Res..

[cit13] Ahmadi A., Khalili M., Nafarie A., Yazdani A., Nahri-Niknafs B. (2012). Mini-Rev. Med. Chem..

[cit14] Guo J., Tao H., Alasadi A., Huang Q., Jin S. (2019). Eat. Weight Disord..

[cit15] Waszkielewicz A. M., Kubacka M., Panczyk K., Mogilski S., Siwek A., Glich-Lutwin M., Grybos A., Filipek B. (2016). Bioorg. Med. Chem. Lett..

[cit16] Petrov V. F., Pavluchenko A. I. (2003). Mol. Cryst. Liq. Cryst..

[cit17] Zhang M., Chen C., Wang Q., Fu W., Huang K., Zhou W. (2017). J. Mater. Chem. A.

[cit18] Safko J. P., Kuperstock J. E., McCullough S. M., Noviello A. M., Li X. B., Killarney J. P., Murphy C., Patterson H. H., Bayse C. A., Pike R. D. (2012). Dalton Trans..

[cit19] Chaudhari A., Kuwar A., Mahulikar P., Hundiwale D., Kulkarni R., Gite V. (2014). RSC Adv..

[cit20] Watson S., Nie M., Wang L., Stokes K. (2015). RSC Adv..

[cit21] Yan L., Wang Y., Wei J., Ji G., Gu H., Li Z., Zhang J., Luo Q., Wang Z., Liu X., Xu B., Wei Z., Ma C. Q. (2019). J. Mater. Chem. A.

[cit22] Naarmann H., Pohlemann H. (1977). Ger. Offen..

[cit23] Gettys E. K., Ye Z., Dai M. (2017). Synthesis.

[cit24] Hall Jr H. K. (1963). J. Org. Chem..

[cit25] Vysochin V. I., Shishkin G. V. (1982). Chem. Heterocycl. Compd..

[cit26] Maras N., Polanc S., Kocevar M. (2012). Org. Biomol. Chem..

[cit27] Somei M., Sayama S., Naka K., Shinmoto K., Yamada F. (2007). Heterocycles.

[cit28] Ross S. D., Finkelstein M. (1963). J. Am. Chem. Soc..

[cit29] Lutze G., Graubaum H., Bartoszek M., Griindemann S., Flatau S. (1996). J. Prakt. Chem..

[cit30] Kolesinska B., Barszcz K., Kaminski Z. J., Drozdowska D., Wietrzyk J., Switalska M. (2012). J. Enzyme Inhib. Med. Chem..

[cit31] Wang H. J., Earley W. G., Lewis R. M., Srivastava R. R., Zych A. J., Jenkins D. M., Fairfax D. J. (2007). Tetrahedron Lett..

[cit32] Wang H. J., Wang Y., Csakai A. J., Earley W. G., Herr R. J. (2009). J. Comb. Chem..

[cit33] Gladstone S. G., Earley W. G., Acker J. K., Martin G. S. (2009). Tetrahedron Lett..

[cit34] Zhu Q., Yuan Q., Chen M., Guo M., Huang H. (2017). Angew. Chem., Int. Ed..

[cit35] Zhu Q., Yang P., Chen M., Hu J., Yang L. (2018). Synthesis.

[cit36] Reddy N. D., Elias A. J., Vij A. (1999). J. Chem. Soc., Dalton Trans..

[cit37] Vrantza D., Kaloudis P., Leondiadis L., Gimisis T., Vougioukalakis G. C., Orfanopoulos M., Gasparutto D., Cadet J., Encinas S., Paris C., Miranda M. A. (2006). Helv. Chim. Acta.

[cit38] Min G., Seo J., Ko H. M. (2018). J. Org. Chem..

[cit39] Ross S. P., Hoye T. R. (2018). Org. Lett..

[cit40] Ross S. P., Hoye T. R. (2017). Nat. Chem..

[cit41] Boursalian B., Ham W. S., Mazzotti A. R., Ritter T. (2016). Nat. Chem..

[cit42] Yavari I., Bayat M. J., Ghazanfarpour-Darjani M. (2014). Tetrahedron Lett..

[cit43] Ghazanfarpour-Darjani M., Barat-Seftejani F., Khalaj M., Mousavi-Safavi S. M. (2017). Helv. Chim. Acta.

[cit44] Bugaenko D. I., Yurovskaya M. A., Karchava A. V. (2017). J. Org. Chem..

[cit45] Mosca L., Rotili D., Tempera I., Masci A., Fontana M., Chiaraluce R., Mastromarino P., d'Erme M., Mai A. (2001). ChemMedChem.

[cit46] Bugaenko D. I., Yurovskaya M. A., Karchava A. V. (2018). Org. Lett..

[cit47] Koyioni M., Manoli M., Koutentis P. A. (2016). J. Org. Chem..

[cit48] Dong H. R., Chen Z. B., Li R. S., Dong H. S., Xie Z. X. (2015). RSC Adv..

[cit49] Hartung J., Hunig S., Kneuer R., Schwarz M., Wenner H. (1997). Synthesis.

[cit50] Fu Y., Xu Q. S., Li Q. Z., Li M. P., Shi C. Z., Du Z. (2019). ChemistryOpen.

[cit51] Phakhodee W., Wangngae S., Pattarawarapan M. (2016). RSC Adv..

[cit52] Boyer J. H., Pillai T. P. (1985). J. Chem. Soc., Perkin Trans. 1.

[cit53] Sayo H., Hosokawa M., Lee E., Kariya K. (1988). Chem. Pharm. Bull..

[cit54] Wu H. Q., Yang K., Chen X. Y., Arulkumar M., Wang N., Chen S. H., Wang Z. Y. (2019). Green Chem..

